# Native and bone marrow-derived cell mosaicism in gastric carcinoma in *H. pylori*-infected p27-deficient mice

**DOI:** 10.18632/oncotarget.12049

**Published:** 2016-09-15

**Authors:** Songhua Zhang, Woojin Kim, Tu T. Pham, Arlin B. Rogers, Jean Marie Houghton, Steven F. Moss

**Affiliations:** ^1^ Division of Gastroenterology, Rhode Island Hospital, Warren Alpert Medical School of Brown University, Providence, RI, USA; ^2^ Department of Biomedical Sciences, Tufts Cummings School of Veterinary Medicine, Tufts University, North Grafton, MA, USA; ^3^ Department of Medicine and Cancer Biology, Division of Gastroenterology, University of Massachusetts Medical School, Worcester, MA, USA

**Keywords:** Helicobacter pylori, gastric cancer, bone marrow transplantation, inflammation, cytokines

## Abstract

**Objective:**

Chronic *Helicobacter pylori* (*H. pylori*) infection promotes non-cardia gastric cancer. Some mouse models suggest that bone marrow derived cells (BMDC) contribute to *Helicobacter*-associated gastric carcinogenesis. We determined whether this increased susceptibility to *Helicobacter*-induced gastric carcinogenesis of p27-deficient mice is dependent upon their p27-null BMDC or their p27-null gastric epithelial cells.

**Design:**

Female mice (recipients) were irradiated and transplanted with BMDC from male donors. Wild type (WT) mice in group 1 (control) received BMDC from male GFP-transgenic mice. Female WT and p27 KO mice were engrafted with male p27KO mice BMDC (Group 2) or GFP-transgenic WT BMDC (Group 3). Recipients were infected with *H. pylori* SS1 for one year.

**Results:**

Mice lacking p27 in either the BM pool or gastric epithelium developed significantly more advanced gastric pathology, including high-grade dysplasia. Co-staining of donor BMDC in dysplastic gastric glands was confirmed by immunofluorescence. Gastric expression of IL-1 beta protein was reduced in groups 2 and 3 (*p* < 0.05 *vs* control) whereas expression of IFN-γ and chemokines MIP-1 beta, MIG, IP-10 and RANTES in group 2 were significantly higher than group 3.

**Conclusions:**

Both bone marrow-derived and gastric epithelial cells contribute to the increased gastric cancer susceptibility of p27-deficient *H. pylori*-infected mice.

## INTRODUCTION

Gastric cancer is the fifth most common neoplasm and the third leading cause of cancer death worldwide. [1] Most cases of gastric cancer are attributable to persistent chronic infection by *Helicobacter pylori* (*H. pylori*), including almost 90% of cases of non-cardia gastric cancer. [2] Since *H. pylori* was designated a definite gastric carcinogen in 1994, [3] the pathogenesis of *H. pylori* infection has been extensively studied, but exactly how this bacterium and/or the associated gastric inflammatory response contributes to gastric carcinogenesis remains poorly defined.

Gastric malignancy usually develops after a multi-step, decades-long process, as proposed by Correa in 1975. [4] In this “conventional model”, the epithelial cells and glands of the gastric mucosa undergo progressive histological change, as confirmed by numerous murine studies. [5–7] Additional steps to this model were proposed by Houghton *et al* in 2004, [8] based upon *H. felis* infection in wild type C57BL/6 mice. In that model, chronic *Helicobacter* infection triggered the migration of bone marrow-derived cells (BMDC) to the stomach, where they gave rise to neoplastic gastric epithelial cells and progressed to gastric adenocarcinoma via the classic changes described by Dr. Correa. [8] *H. pylori* infection in mice induces less severe gastric mucosal alteration, and rarely if ever causes adenocarcinoma. Nevertheless, support for the “BMDC model” has come from similar experiments by Varon and colleagues using mice infected by *H. pylori* Sydney Strain 1 (SS1), [9] in which BMDC were identified in approximately 25% of dysplastic gastric lesions, which are thought to be precursors of gastric adenocarcinoma.

Since wild type C57BL/6 mice rarely develop gastric cancer, even after 80 weeks *H. pylori* infection, [10] our goal was to evaluate the contribution of BMDC to gastric cancer in a mouse model that is susceptible to *H. pylori*-induced gastric carcinogenesis. [11] For the current study, we chose p27^Kip1^ deficient (p27 KO) mice, since our laboratory has shown that these mice develop gastric cancer about a year after *H. pylori* SS1 infection, after slowly progressing through a sequence of pathological changes, recapitulating human *H. pylori*-associated gastric carcinogenesis. [12]

We used the p27-deficient gastric cancer prone model to evaluate the relative contributions of BMDC to epithelial cells in *H. pylori*-induced gastric cancer. BMDC are critical for the development of dysplasia and neoplasia in the *Helicobacter* models, however, the relative contribution of genetic defects in the epithelial and BM cell compartments have not been studied. We hypothesized that if BMDC were critical for *H. pylori*-induced gastric carcinogenesis in p27-deficient mice, then the p27-deficient bone marrow-derived, rather than the p27-deficient gastric epithelial cells would be responsible for gastric tumorigenesis following *H. pylori* infection. To determine whether the loss of p27 expression in bone marrow-derived cells (in a background of wild type epithelium) or loss of p27 expression in gastric epithelial cells (with wild type BM cells) contributes to in *H. pylori*-infected gastric mucosa, we generated chimeric mice by transplantation of BM via lateral tail vein injection of p27 knock-out or wild type BMDC into wild type and p27-deficient recipients, respectively, using wild type to wild type autologous mice to control for the effects of radiation and bone marrow transplantation. After bone marrow transplantation and long term *H. pylori* infection, we evaluated cell lineages in the context of gastric pathological changes to address the cellular origins of *H. pylori*-associated gastric carcinoma.

## RESULTS

There were three groups of mice in the present study, all on a C57BL/6 background, as shown in Table 1. All donors were males and recipients were females.

**Table 1:** Experimental Design

	Donors	Recipients
Group 1 (Control)	♂ Wild Type (GFP-expressing)	♀ Wild Type
Group 2	♂ p27 −/− Mice	♀ Wild Type
Group 3	♂ Wild Type (GFP-expressing)	♀ p27−/− Mice

Donor and recipient mice in three experimental groups. In this study, all of the mice have C57BL/6 background and all donors are males, recipients are females. ♂ : Male, ♀ : Female, GFP: Green Fluorescent Protein.

### Successful bone marrow transplantation

**Figure 1:**
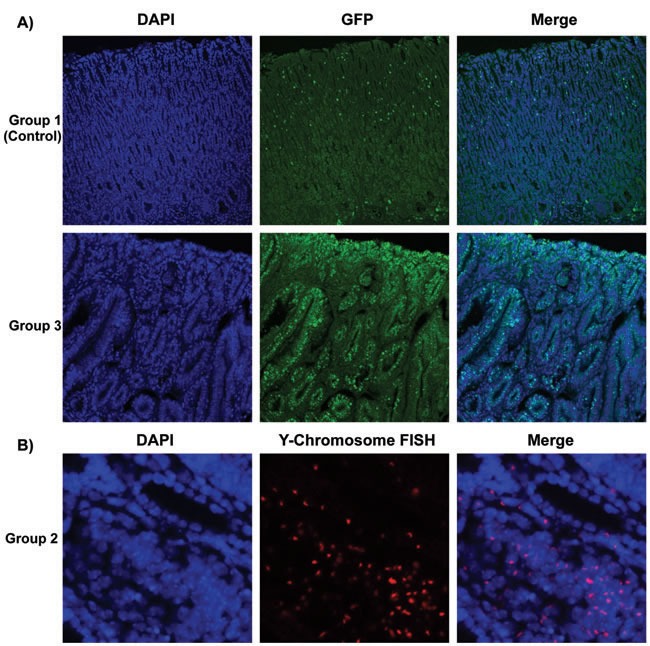
Determination of Bone Marrow-derived GFP-expressing Cells And Male Donor-derived Y-chromosome in Recipient Mice infected by *H. pylori* **A.** Immunofluorescence staining detected marrow-derived cells, which were stained green in group 1 and group 3. Nuclei labeled blue by DAPI in left panels. **B.** In group 2, Y-chromosomes from male donor mice were detected by performing Fluorescence in situ hybridization (FISH). Donor derived Y-chromosomes were labeled with Cy3-conjugated probe (red), which were located within the DAPI stained nucleus (blue) in merged images. Many of the donor cells were incorporated in gastric glands.

For the mice that recovered from sublethal irradiation, bone marrow transplantation was evaluated 52 weeks after *H. pylori* infection as shown in Figure 1. Since the bone marrow donor mice in experimental groups 1 and 3 expressed enhanced green fluorescent protein (GFP), immunofluorescence staining was performed to detect these bone marrow-derived cells (stained green) in the stomachs of recipient mice (Figure 1A). In the mice in experimental group 2, the presence of donor bone marrow-derived cells in the stomachs of the recipients was instead confirmed by staining Y-chromosomes from the male donors by FISH. Donor derived Y-chromosomes were labeled with a Cy3-conjugated probe (red), located within the DAPI stained nucleus (blue) in merged image (Figure 1B). The presence of bone marrow-derived GFP-positive cells (group 3) or Y-chromosome stained cells (group 2) could be frequently detected in the dysplastic gastric glands in these two experimental groups of mice (Figure 1).

### Histological evaluation

**Figure 2:**
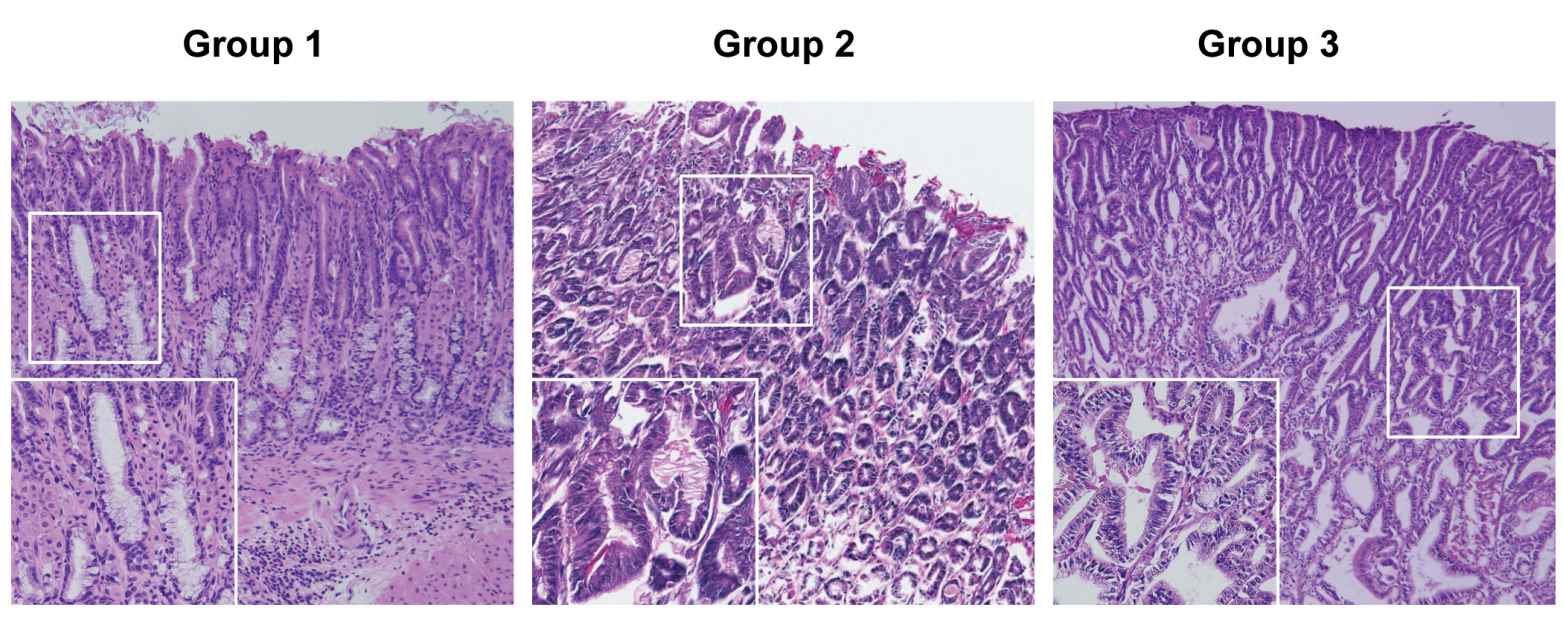
Representative Gastric Histopathology in Recipient Mice Representative histopathology from the three groups of mice at 52 weeks post *H. pylori* infection (Hematoxylin and eosin staining). Mice in the control group (Group 1) developed mild gastric epithelial hyperplasia and mild pseudopyloric metaplasia but, unlike the mice in groups 2 and 3, there was no progression to dysplasia. In contrast there was evidence of more advanced gastric pathological abnormality in mice in groups 2 and 3. Original magnification x 100, higher power (insets) x 200.

Mice were euthanized 52 weeks after *H. pylori* infection by gavage (all groups), and the gastric histology was evaluated by hematoxylin and eosin (H&E) staining. Representative H&E stained histological images from the three groups at 52 weeks post infection are shown in Figure 2, with higher magnification images in the left lower corner of each panel. In the control group (Group 1), all the mice survived until the end of the study. Although our aim was to determine the origin of gastric cancer cells, we did notice differences in survival curves during the study. In Kaplan-Meier analysis, group 3 p27 KO mice which received wild type bone marrow had significantly poorer overall survival compared with group 1 control mice and group 2 wild type mice which received p27 KO marrow cells (data not shown). The cause of premature death in our group 3 mice was likely from multiple endocrine and/or hematological tumors rather than their stomach pathology. [13–15]

**Figure 3:**
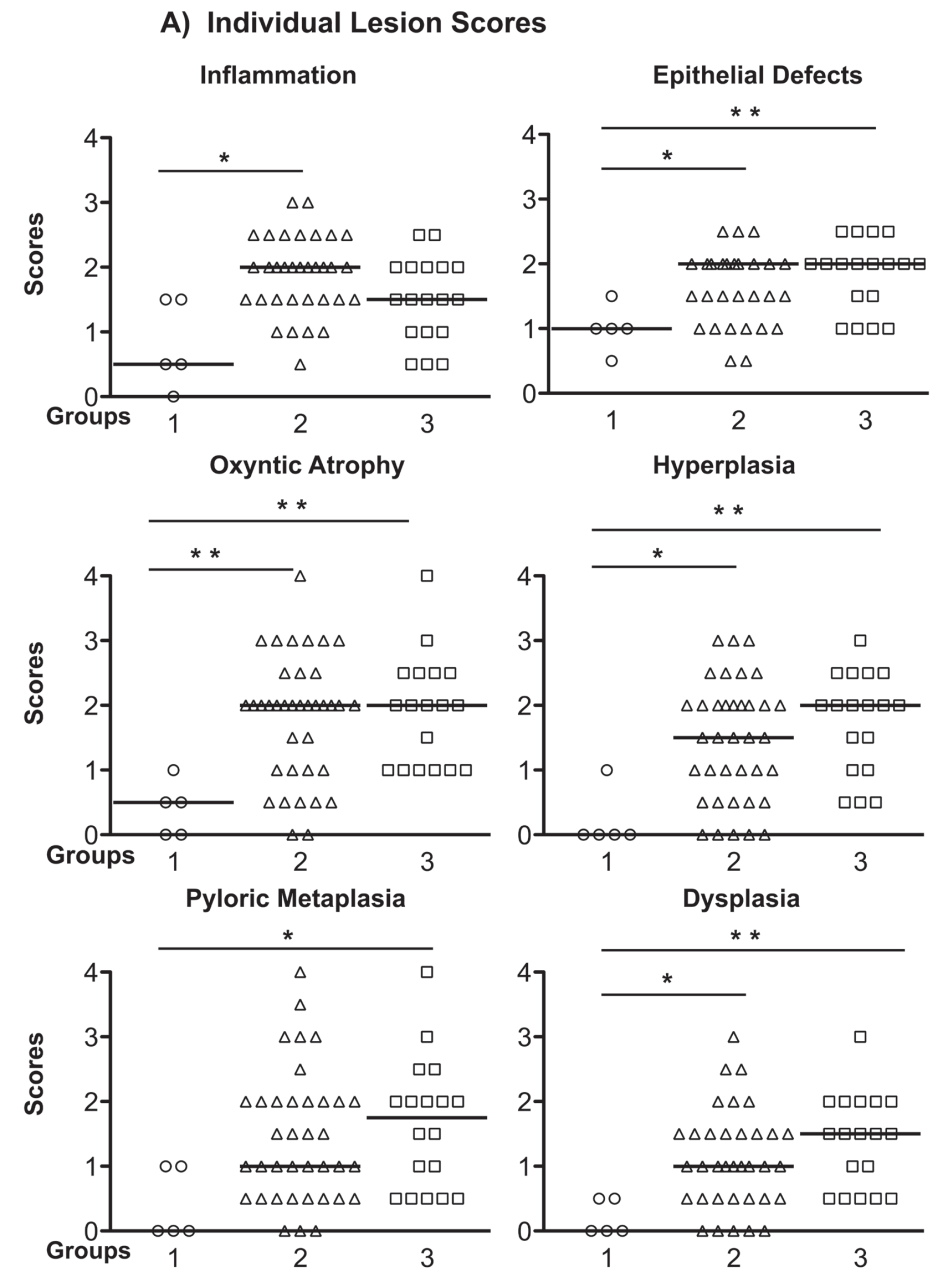

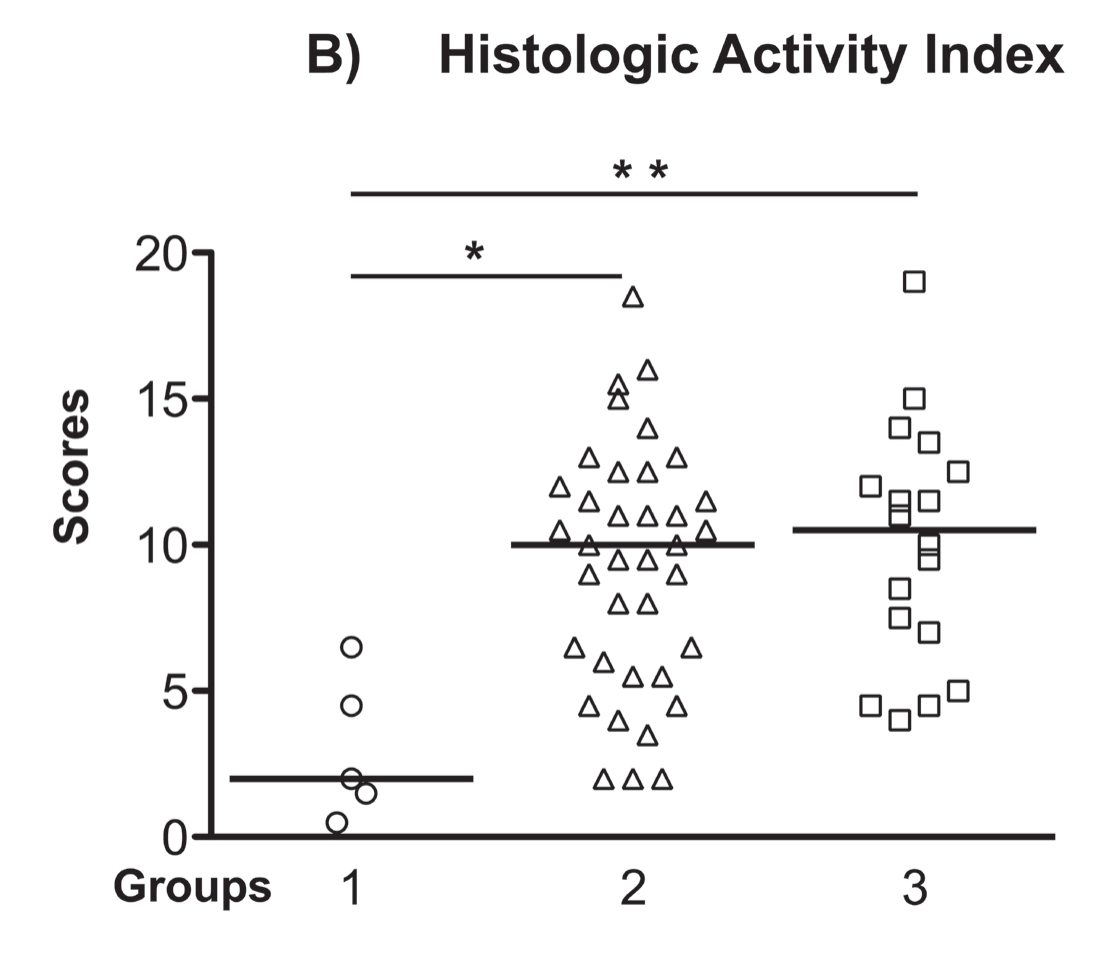
Gastric Histology Scores in *H. pylori*-infected Recipient Mice **A.** The Individual gastric lesion scores of every mouse in each group, including inflammation, epithelial defects, oxyntic atrophy, hyperplasia, pyloric metaplasia, and dysplasia, were graded on a scale of 0 - 4. **B.** Total Histological Activity Index is a combination of all the six individual criteria scores (scored from 0 - 24). In general, wild type mice in the control group showed a milder gastric pathology, while the two other groups developed advanced pathological lesions. Each empty circle, triangle and square represents one mouse in each group, respectively. The bar represents the median score in each group. *N* = 5, 37, 18 in groups 1, 2, 3, respectively. *P < 0.05, **P < 0.01 *vs* group 1 (control). There was no statistically significant difference between group 2 and group 3 mice.

Although group 1 mice have intensive gastric inflammatory cell infiltration, they retained normal gastric epithelial architecture, and only developed mild hyperplasia and pseudopyloric metaplasia (higher magnification image), but no dysplasia. In contrast, mice in groups 2 and 3 exhibited more complex gastric mucosal architecture with dysplastic features including atypical and heterogeneous gastric glands, coupled with variability in epithelial cell size and enlarged and hyperchromatic nuclei (see higher magnification image). These histopathological findings were quantified for each animal (Figure 3) by scoring inflammation, epithelial defects, oxyntic atrophy, hyperplasia, pyloric metaplasia, and dysplasia separately on a scale of 0 – 4 and comparing differences among groups (Figure 3A). [11] The total Histologic Activity Index (Figure 3B), a combination of the above individual criteria scores, was also calculated. In general, wild type (WT) mice in the control group showed a milder gastric phenotype for all parameters, whereas mice in groups 2 and 3 developed statistically significantly more severe lesions across criteria. The statistical analysis showed no significant differences among the scores for group 2 and group 3 mice. None of the mice developed invasive cancer.

### Bone marrow-derived cells express E-cadherin and incorporate into gastric glands

**Figure 4:**
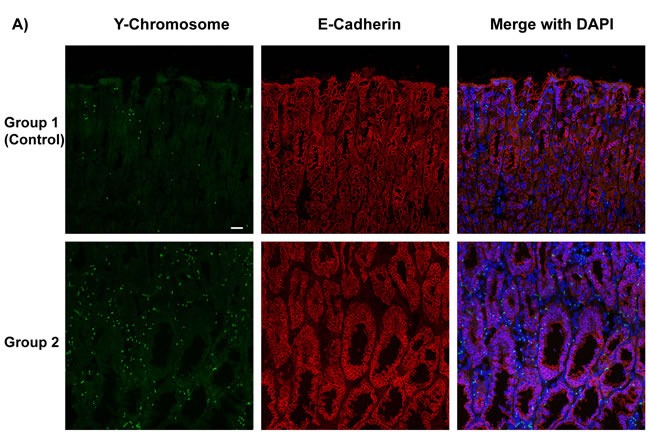

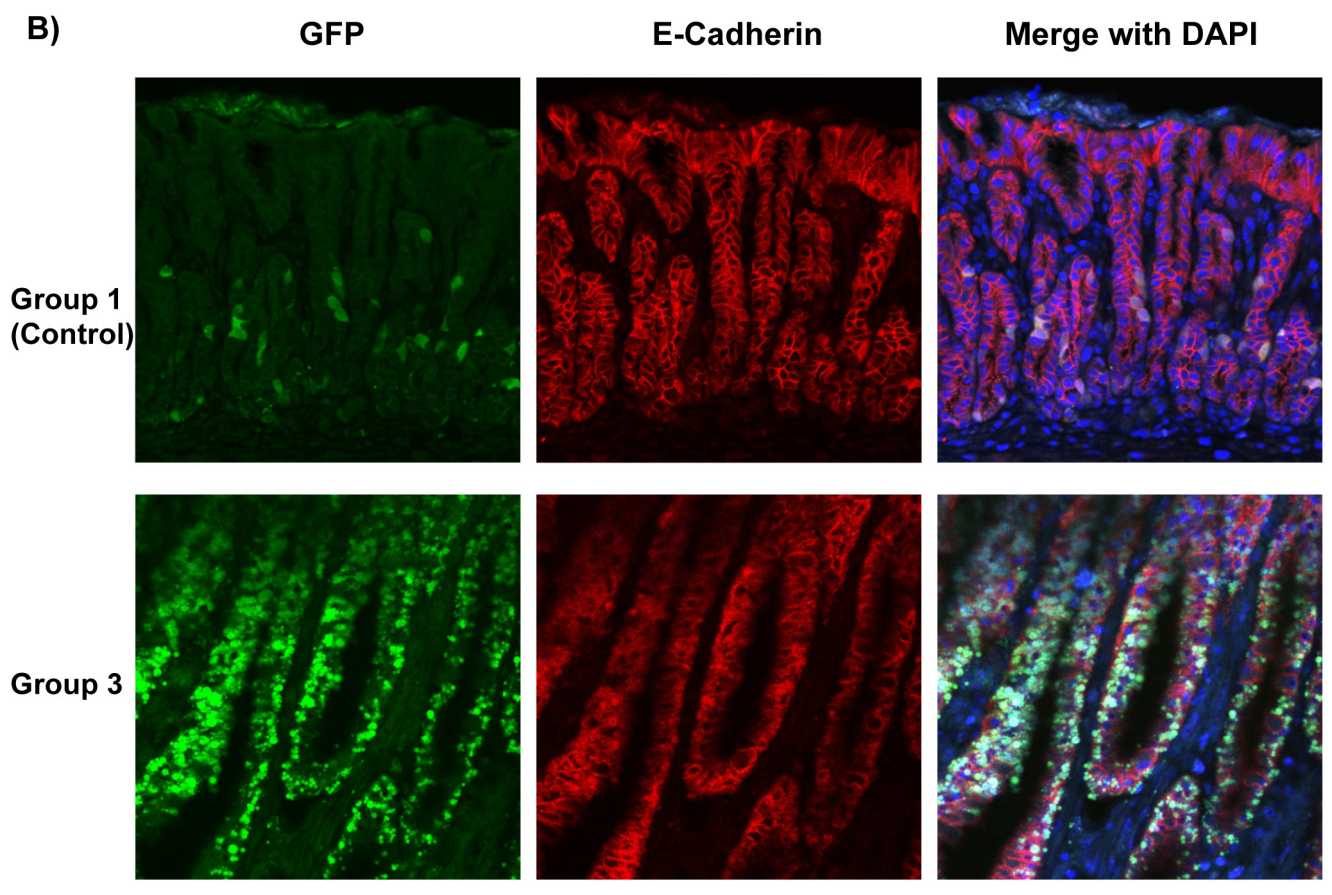

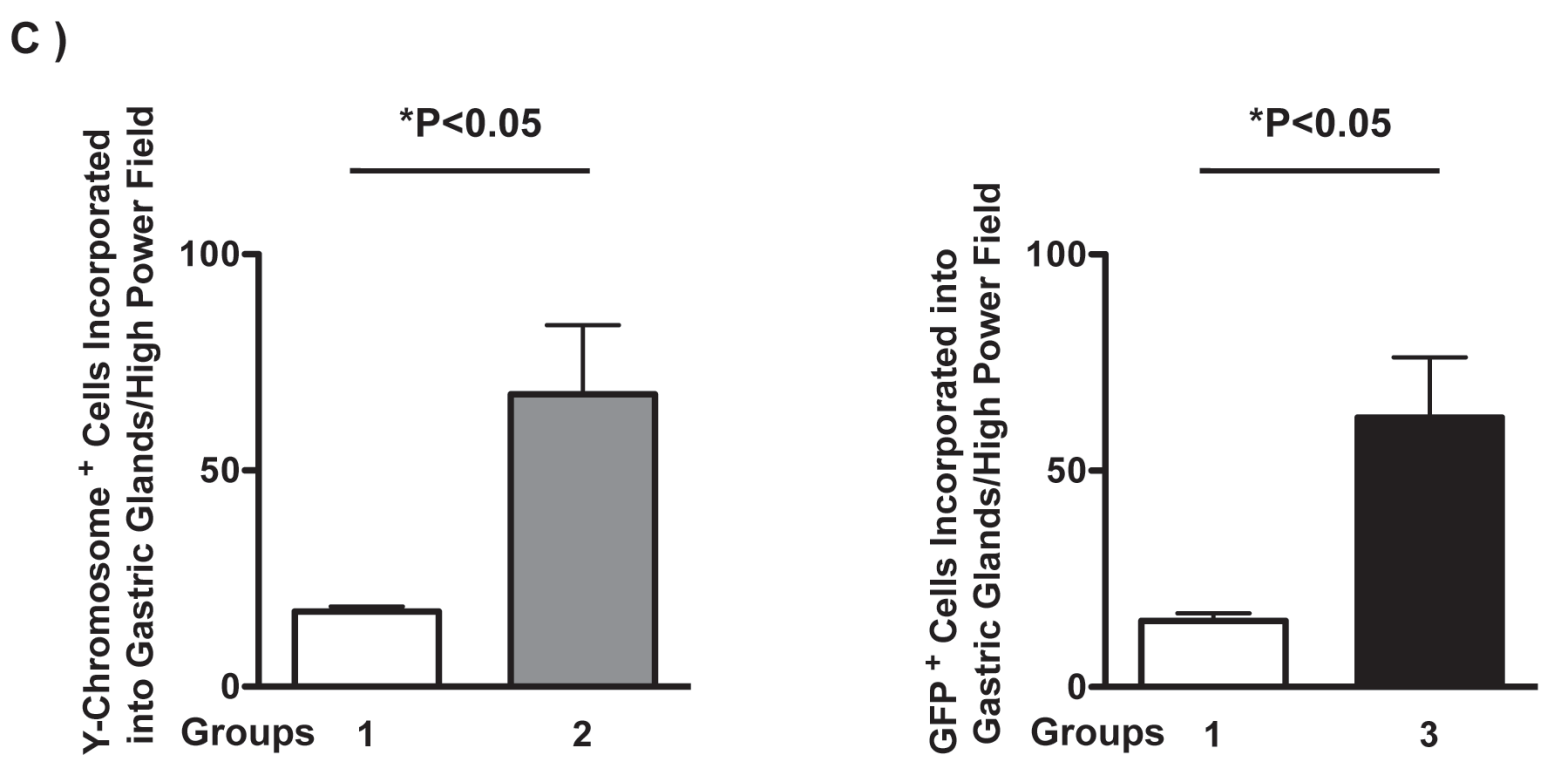
Bone Marrow-Derived Cells (BMDCs) Incorporated into Gastric Glands **A.** Y-Chromosome detection by FISH (green) was combined with E-Cadherin (red) immunofluorescence staining, then merged with DAPI (blue) in groups 1, 2. Bar: 20 μm. **B.** GFP (green) and E-Cadherin (red) immunofluorescence staining were merged with DAPI (blue) in groups 1 and 3. These images clearly showed that BMDCs incorporated into gastric glands. BMDCs were frequently observed in dysplastic gastric glands in group 2 and 3. **C.** Left panel: Quantification of Y-Chromosome positive BMDCs incorporated into gastric glands per high power field. *P < 0.05. Right panel: Quantification of GFP positive BMDCs incorporated into gastric glands per high power field. *P < 0.05. GFP: Green Fluorescent Protein.

Cadherins are important calcium-dependent cell transmembrane adhesion proteins present on gastric epithelial cells; downregulation of E-cadherin has been implicated in tumorigenesis. [16] As group 2 mice received BMDC from p27 deficient mice, no GFP-expressing cells were detectable. To detect whether bone marrow-derived cells in mice express E-cadherin and incorporate into gastric glands, we performed triple-color staining in each group. We conducted E-Cadherin (red) immunofluorescence staining and combined it with either Y-Chromosome FISH in groups 1 and 2, or immunofluorescence staining for GFP (green) in groups 1 and 3. Subsequently, nuclei were stained with DAPI (blue) to provide composite images (Figure 4A and 4B). The staining clearly showed male donor-derived BMDC incorporated into gastric glands. Gastric epithelial cells were marked with E-Cadherin. Bright, intact E-Cadherin staining was seen in the control group whereas there was much weaker and discontinuous E-Cadherin expression in dysplastic areas in group 2 and 3 mice. Y-chromosome positive or GFP-expressing donor mouse derived BMDCs were abundant in *H. pylori*-infected gastric mucosa and in the dysplastic gastric glands of group 2 and 3, while only very few BMDCs in group 1 (control) were detected. In group 3 mice, there were areas of numerous GFP-positive cells in some irregular dysplastic glands, with very few non-donor-derived cells. And within the glands, these donor cells also expressed E-cadherin (Figure 4A and 4B).

In comparison with control mice, donor-derived Y-chromosome positive cells were more frequent in group 2 mice (Figure 4C, left panel, 17 ± 1 *vs* 68 ± 16 per high power field, *P < 0.05). More GFP-expressing BMDCs were engrafted in the inflamed gastric mucosa and incorporated into the gastric glands, especially in dysplastic glands, in group 3 mice compared with the control (group 1 mice) (Figure 4C, right panel, 15 ± 2 *vs* 62 ± 14 per high power field, *P < 0.05). Data were expressed as mean ± standard error of the mean (SEM).

### Expression of proinflammatory cytokines and chemokines in gastric mucosa

**Figure 5:**
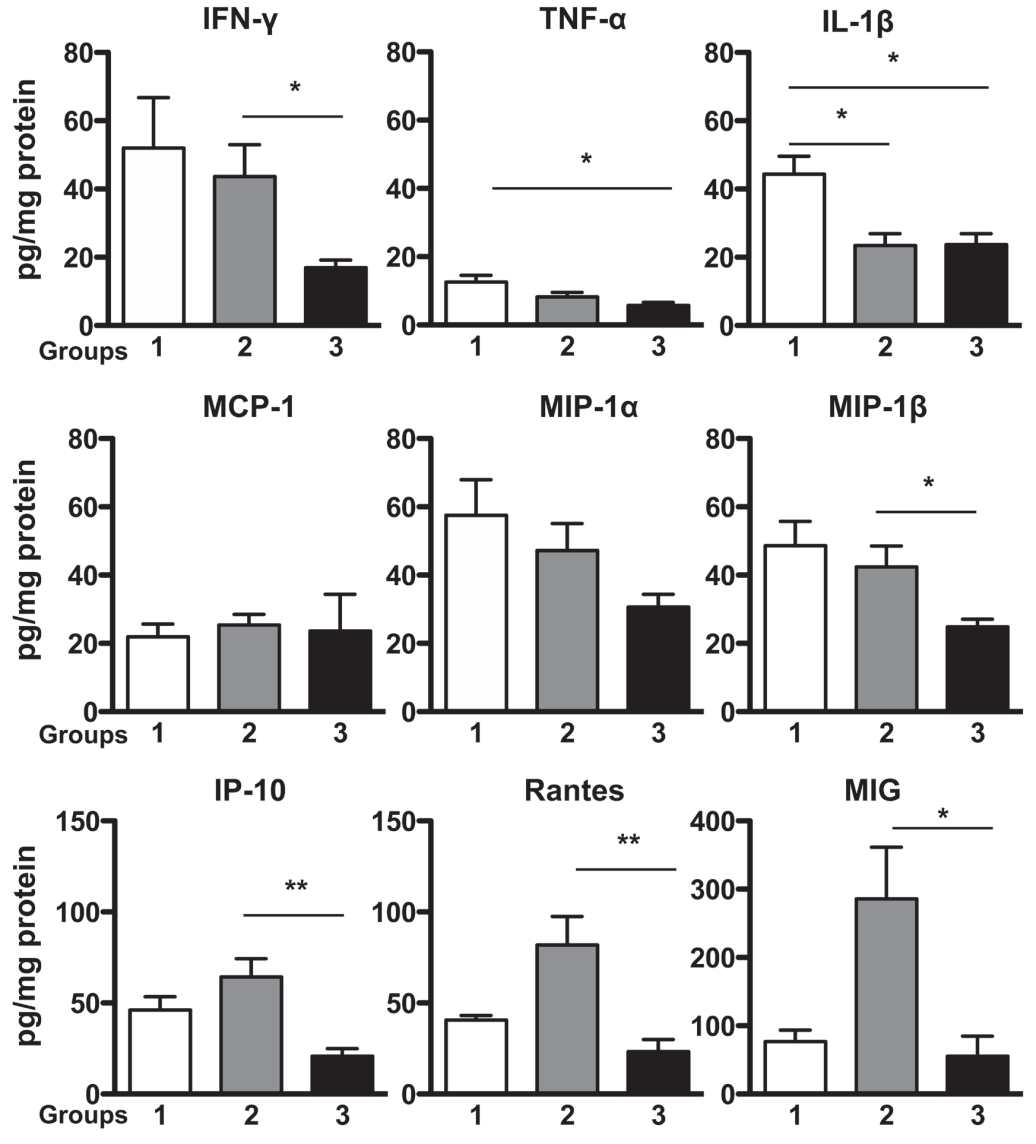
Expression of Proinflammatory Cytokines and Chemokines in Gastric Mucosa Protein levels of specific proinflammatory cytokines and chemokines in mice stomach tissue were analyzed in a multiplex assay. There were no significant differences in protein levels of MCP-1 and MIP-1 α among the three groups, while IFN-γ, MIP-1 β, IP-10, RANTES and MIG levels were significantly elevated in group 2 when compared with group 3. TNF-α levels in group 3, and IL-1β expression in group 2 and group 3 were significantly lower than those protein levels in control group, respectively. Data were expressed as mean ± SEM (pg/mg protein). SEM: the standard error of the mean. *N* = 5, 22, 18 in groups 1, 2, 3, respectively. *P < 0.05, **P < 0.01 *vs* control.

To evaluate the nature of the inflammatory milieu we assessed the gastric mucosal levels of three proinflammatory cytokines and six chemokines using a customized multiplexed protein quantification assay, according to the manufacturer's instructions (EMD Millipore, MA, USA). Data were expressed as mean ± SEM. There were no significant differences in protein levels of MCP-1 and MIP-1 α among the three groups. However, in comparison with controls, TNF-α levels were reduced in group 3 (5.75 ± 0.85 pg/mg protein, *P* < 0.05 *vs* control 12.61 ± 1.93 pg/mg protein), and there was lower IL-1β expression in groups 2 and 3 (23.45 ± 3.48 pg/mg protein; 23.75 ± 3.18 pg/mg protein, respectively, *P* < 0.05 *vs* control 44.37 ± 5.23 pg/mg protein). When compared with group 3 mice, IFN-γ (43.62 ± 9.36 pg/mg protein) and MIP-1 β expression (42.39 ± 6.19 pg/mg protein) in group 2 were markedly elevated (*P* < 0.05 *vs* 16.91 ± 2.27 pg/mg protein; 24.83 ± 2.26 pg/mg protein in group 3, respectively). In addition, IP-10, RANTES and MIG levels were significantly increased in group 2 (64.38 ± 9.94; 81.79 ±15.82; 286.1 ± 75.39 pg/mg protein, when compared with group 3 (20.84 ± 4.1; 23.21 ± 6.79; 55.31 ± 29.4 pg/mg protein, respectively, *P* < 0.05 *vs* group 3 for each comparison). *N* = 5, 22, 18 in groups 1, 2, 3, respectively (Figure 5).

## DISCUSSION

Chronic *H. pylori* infection is a major risk factor for the development of distal gastric carcinoma. [17] Despite decades of research in this field, how *H. pylori* and its associated gastric inflammatory response promote gastric neoplasia remains unclear. Studies in mouse models using *H. felis*, a murine-adapted *Helicobacter*, supports the notion that the cancer stem cell in gastric cancer originates from bone marrow-derived cells drawn to an inflamed gastric milieu by chronic bacterial colonization. [8, 9] Similar findings have been reported using *H. pylori*, lending more human relevance to the findings. However, *H. pylori* infection in the mouse model progresses to dysplasia and not to frank carcinoma. Therefore, it remains uncertain whether gastric epithelial cells or bone marrow-derived stem cells initiate gastric cancer development in the *H. pylori*-infected mouse model.

In the present study, we utilized cancer-prone p27-deficient mice that develop gastric cancer about one year after experimental *H. pylori* infection [12] to explore whether their increased cancer susceptibility was due to p27 deficiency in their bone marrow-derived cells or in their gastric epithelial cells. Wild type mice receiving autologous bone marrow followed by *H. pylori* infection served as an experimental control to evaluate the effects of radiation, bone marrow transplantation, and to compare with the other two groups in terms of pathological changes and cytokine expression. Using GFP and Y-chromosome markers to track bone marrow-derived cells, we observed that very few GFP+ bone marrow-derived cells were incorporated into the normal gastric glands of the control mice whereas many cells derived from the bone marrow appeared to be incorporated into the dysplastic gastric glands of mice deficient in p27. Image quantification analysis further confirmed that compared with group 1 control mice, there was a higher frequency of Y-chromosome positive or GFP-expressing donor mouse derived BMDCs incorporated into the recipient's gastric glands, especially within the dysplastic areas of group 2 and 3 mice. These results indicate that the irradiation protocol does not cause significant structural damage in gastric mucosa, and that engrafted bone marrow cells participate in the development of gastric dysplasia related to *H. pylori* stimulation. These results therefore support previous findings in different *Helicobacter*-infected mouse models. [8, 9]

Since our previous reports demonstrated that *H. pylori*-infected p27-deficient mice are susceptible to advanced gastric preneoplastic and neoplastic lesions, [12, 18] we predicted that if gastric cancer originated in bone marrow-derived cells, then wild type mice that received p27-deficent bone marrow cells (group 2) should have the highest cancer incidence of the three experimental mouse groups. However, our results indicated that the increased susceptibility of these mice to *H. pylori*-induced gastric pathology was equal to that of the mice in group 3 that lacked p27 in all cells except the bone marrow.

Group 2 mice, which received p27-deficient bone marrow, showed significantly higher inflammation scores compared with control mice. Although p27 deficiency does not increase the number of stem cells in the bone marrow, mice lacking p27 have increased cytokine-stimulated T cell proliferation in the peripheral lymphoid organs, [19–21] which may lead to increased cytokine secretion and enhanced inflammatory responses in the stomachs of the wild type *H. pylori-*infected mice that received p27-deficient bone marrow cells. Mice in group 3 that lacked p27 in all cells *except* their bone marrow cells also displayed increased inflammation compared with autologous wild type controls, though the difference was not statistically significant, probably because of a small sample size in the controls. Nevertheless, this indicates that gastric epithelial cell p27 may normally play a role in down-regulating inflammatory and pro-neoplastic responses to *H. pylori*.

Cytokines and chemokines are important mediators that mediate and coordinate host immune responses to pathogen infection and invasion. Previous studies have demonstrated that *H. pylori* infection induces the expression of multiple inflammatory cytokines and chemokines. [22–24] We investigated the nature of gastric inflammatory during chronic *H. pylori* infection in all groups of mice in our study through sampling the expression of several proinflammatory cytokines (IFN-γ, TNF-α and IL-1β) and chemotactic chemokines (MCP-1, MIP-1α, MIP-1β, IP-10, RANTES and MIG) by a protein multiplex array. At 52 weeks after *H. pylori* infection, there was very low expression of the T helper 1 cytokine TNF-α, and no significant differences in MCP-1 and MIP-1α among the three groups. Unexpectedly, in contrast to other studies, IL-1β expression in groups 2 and 3 that developed gastric dysplasia was significantly lower than that of control group. [25, 26]

Interestingly, although the severity of mucosal lesions and the degree of inflammation assessed by standard pathological criteria were similar in group 2 and group 3 mice, they displayed quite different cytokine/chemokine protein expression, with significantly increased protein levels of IFN-γ, MIP-1β, IP-10, RANTES and MIG in group 2 mice. MIP-1β and RANTES are members of the CC chemokine family, and are homologous to their human CCL4 and CCL5 counterparts, respectively. Both these CC cytokines can bind to CCR5 receptor and act as potent chemoattractants. CCL5 is also involved in wound healing [27]. Recent studies have showed that RANTES is associated with gastric cancer progression, [28, 29] and increased expressions of IP-10 and MIG have also been implicated in gastric cancer. [30, 31] IP-10 and MIG, binding to the same CXCR3 receptor, are IFN-γ related chemokines, and they usually increase in parallel during inflammation. [32–34] Our prior experiments in p27-deficient mice support a role for markedly elevated IP-10 and MIG protein levels in the dysplastic gastric mucosa consequent to persistent *H. pylori* infection. [18] These elevated cytokines and chemokines in group 2 mice that received p27 deficient bone marrow cells support the idea that BMDC are recruited to the gastric mucosa by increased production of chemoattractants locally that may serve to perpetuate ongoing inflammation. Control and group 3 mice that received wild type bone marrow showed a similar and relatively low level of IP-10, RANTES and MIG, compared with mice in group 2. In contrast to prior experimental bone marrow transplantation in the context of *H. pylori* infection, [8, 9] group 3 mice that were lacking p27 in all cells except in their bone marrow displayed a high frequency of bone marrow-derived inflammatory cells incorporated into their dysplastic glands. However, since the gastric cytokine and chemokine profiles of these mice were generally similar to that observed in the control mice, which suggests that the mechanisms driving bone marrow cell recruitment to the gastric mucosa and incorporation of BMDC into dysplastic glands must be distinct from the CC/IP-10/MIG-associated processes underlying the carcinogenesis in group 2 mice.

A possible limitation of this study as it applies to human *H. pylori*-induced gastric carcinogenesis is the use of a single mouse-adapted *H. pylori* strain, SS1, that is known to lose *cag* pathogenicity island functionality *in vivo* [35] We used SS1 because it is the standard *H. pylori* strain for mouse models [36] and because it is known to induce high-grade dysplasia in p27 knock out mice in our laboratory. [12] However, we appreciate that additional effects related to the translocation of cagA, an important virulence factor for human gastric cancer development, may also be clinically relevant.

In conclusion, our results demonstrate that both bone marrow-derived and gastric epithelial cells contribute to the increased gastric cancer susceptibility of p27-deficient *H. pylori*-infected mice. Based on the results from three experimental groups in the present study, we conclude that both BMDC and gastric epithelial cells are involved in the development of gastric neoplasia following *H. pylori* infection, acting in concert but through two distinct pathways. Clinical and translational studies to address the relevance of these findings to human gastric carcinogenesis should now be considered.

## MATERIALS AND METHODS

### Mice, bone marrow transplantation and *H. pylori* infection

Male and female homozygous p27^kip1^-deficient mice were bred in our laboratory from six to eight week-old heterozygous p27-deficient founders purchased from The Jackson Laboratory (Bar Harbor, ME, USA) as described previously. [18] Male C57BL/6-Tg (CAG-EGFP) 131 Osb/LeySopJ mice (Stock number: 006567) were used as bone marrow donors in group one and group 3; female C57BL/6 wild type mice were used as recipients in group one and group 2. All mice were housed in micro-isolator cages under positive air pressure and fed autoclaved laboratory chow and water ad libitum throughout. The protocol was approved by Rhode Island Hospital's Animal Care and Use Committee.

At 6 weeks of age, recipient mice were sub-lethally exposed to two doses of 450 centigray (cGy) on the same day, two hours apart, using a Gammacell^®^ 40 Exactor Irradiator (Best Theratronics, Ottawa, Ontario K2K 0E4, Canada) and reconstituted with 3 x10^6^ bone marrow (BM) cells from GFP transgenic or p27-deficient mice by lateral tail vein injection as previously described. [8]

Four weeks after bone marrow transplantation, after allowing the recipient mice to recover from irradiation, all mice were gavaged with *H. pylori* Sydney Strain (SS1) of approximately 10^9^ bacterial colony forming units (CFU) in (200 μl) volume on three occasions over five days as described previously. [18]

All mice were euthanized and their stomachs were removed 52 weeks after *H. pylori* infection, or sooner if their condition met Rhode Island Hospital's Animal Care and Use Committee criteria for signs of distress. Stomachs were opened along the greater curvature and cut into several linear strips for DNA isolation, protein extraction and histologic analysis. Mouse serum samples were collected for anti-*H. pylori* IgG detection. *H. pylori* infection status was determined by a combination of positive findings on polymerase chain reaction (PCR) of DNA isolated from mouse gastric tissue, and anti-*H. pylori* IgG ELISA, as described previously. [37] Only infected mice were included in the final analyses.

### Y-chromosome detection by fluorescence *in situ* hybridization (FISH), immunofluorescent histochemical staining, and image quantification

Y-chromosomes from male donor mice in group 2 were detected by performing Fluorescence in situ hybridization (FISH). Cyanine (Cy™3) labeled Y-chromosome was detected by following the kit's two-day protocol (Cambio Ltd, Cambridge, UK). In brief, slides were immersed in pre-warmed denaturation buffer (70% formamide in 2x saline sodium citrate buffer, Invitrogen, Carlsbad, CA, USA) for 2-5 minutes at 65 °C and the probe was denatured at 65 °C for 10 minutes. A mixture of probe (2 μl) and hybridization buffer (8 μl) were carefully added to the tissue on each slide. Then the slides were placed in a sealed humidified chamber, and hybridized at 37 °C overnight. After washing, the tissue section was mounted with Vectashield anti-fade mounting medium with 4′, 6-diamidino-2-phenylindole (DAPI) to counterstain nuclei (Vector Laboratories, Burlingame, CA).

Y-chromosome detection by FISH and anti-E-cadherin immunofluorescence staining was also performed on frozen tissue sections of group 1 and 2 mice. The mouse Y-chromosome probe used for this experiment was labeled with Green 5-Fluorescein dUTP (Empire Genomics, Buffalo, NY, USA). Hybridization was carried out according to the manufacture's protocol. Anti-GFP and anti-E-cadherin immunofluorescence staining were performed on paraffin-embedded tissue sections after antigen retrieval and permeabilization of cell membranes. Polyclonal chicken anti-mouse GFP antibody was applied at a dilution of 1:200 (Invitrogen Corporation, Carlsbad, CA, USA), and monoclonal anti-E-cadherin antibody was used at 1:400 (Cell Signaling Technology, Danvers, MA, USA). The slides were incubated overnight at 4°C. FITC-conjugated donkey anti-chicken and Cy™3-conjugated donkey anti-rabbit secondary antibodies (Jackson Immunoresearch Laboratories Inc., West Grove, PA, USA) were applied at 1:400 dilutions for 1 hour at room temperature. All slides were mounted with Vectashield anti-fade mounting medium with DAPI (Vector Laboratories, Burlingame, CA, USA). Images were obtained with either a Nikon C1si confocal microscope or a Zeiss LSM 710 confocal laser-scanning microscope. Identification of Y-chromosome positive cells was based on the presence of bright green spots inside DAPI-stained blue nuclei. Positive GFP cells were detected as bright green staining localized in the cytoplasm.

To quantify the number of Y-chromosome positive BMDCs that were incorporated into gastric glands, we counted such cells in tissue sections from 3 representative mice in each group. Five 8-bit grayscale consecutive high power fields per slide were acquired with a Nikon E800 microscope (Nikon Inc. Mellville, NY, USA) using a 40x Plan Apo objective. Quantitative data were expressed as the mean of donor-derived cells incorporated into the gastric glands ± SEM per high power field for each experimental group.

### Histologic evaluation

Each mouse stomach was opened along the greater curvature and quickly cut into linear strips. Longitudinal gastric strips (including two from the lesser curvature) were fixed in 10% neutral-buffered formalin, embedded in paraffin, and stained with H&E. Gastric mucosal lesions in each mouse were evaluated by a veterinary pathologist who was blinded to the experimental details using the scoring criteria (0 - 4.0) described previously. [11] As mucous metaplasia and hyalinosis may develop spontaneously in mice, [6] these sub-scores were excluded from the calculation of the histological activity index (HAI).

### Proinflammatory cytokines and chemokines measurement

We evaluated a panel of three proinflammatory cytokines and three chemokines at the protein level by a customized multiplex magnetic beads array (EMD Millipore, Billerica, MA, USA). The panel comprised interferon-gamma (IFN-γ), tumor necrosis factor-alpha (TNF-α), interleukin-1 beta (IL-1β), monocyte chemoattractant protein-1 (MCP-1), macrophage inflammatory protein 1-alpha (MIP-1α) and macrophage inflammatory protein 1-beta (MIP-1β), interferon gamma-induced protein 10 (IP-10) regulated upon activation, normal T cells-expressed and secreted chemokine (RANTES), and Monokine induced by gamma interferon (MIG). For these analyses a strip of stomach tissue from each mouse was homogenized and total protein concentration was measured by BCA protein assay kit (Pierce Biotechnology, Rockford, IL). Aliquots of the protein samples were then applied to a customized multiplex magnetic bead array (EMD Millipore, Billerica, MA, USA) according to the manufacturer's instructions. Data were normalized to total protein concentration in each sample and expressed as mean ± SEM pg/mg protein.

### Statistics

All statistical analysis was performed using Graphpad Prism 5.0 software (San Diego, CA) with a *P* level < 0.05 considered as statistically significant. Histopathological scores were compared by Kruskal-Wallis one-way analysis of variance (ANOVA) followed by Dunn's multiple comparison tests. Y-chromosome positive and GFP positive donor-derived cells were compared by student's t-test. Cytokine and chemokine expression were compared with ANOVA followed by Bonferroni's multiple comparison method. All analyses were confined to those mice that were confirmed to be successfully infected by *H. pylori.*
